# Supplementary material for: Weak population structure in the ant Formica fusca
Source: PeerJ. 2018 Jun 19;6:e5024. doi: 10.7717/peerj.5024 (PMC6014328; doi:10.7717/peerj.5024)
Supplement: Supplemental Information 4 [file peerj-06-5024-s004.docx]

**Supplementary Figures**

**Figure S1** L(K) from STRUCTURE analysis, by way of Structure Harvester. Highest L(K) was obtained for *K*=2. The variance over ten runs was rather large, suggesting that the analysis was unstable, with the exception of *K*=1.

**Figure S2** Evanno’s delta K, by way of Structure Harvester. Delta K suggested two populations in the data set.

**Figure S3** Assignment barplot from STRUCTURE analysis for *K*=2. Every individual was assigned in similar proportions (0.40-0.60) to both clusters, indicating panmixia.

Individual assignment probabilities for K=2.


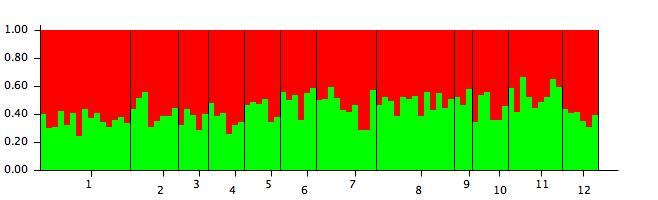


Population
